# Morphologic and Diffusion-weighted MRI Characteristics of Axillary Lymph Nodes for Predicting Metastasis in Breast Cancer Patients: A Quantitative Analysis

**DOI:** 10.2174/0115734056440748260522074819

**Published:** 2026-05-25

**Authors:** Mehmet Sirik, Melahat Poyraz, Ela Kaplan

**Affiliations:** 1 Department of Radiology, Faculty of Medicine, Adıyaman University, Adıyaman, Türkiye; 2 Department of Radiology, Elazığ Training and Research Hospital, Elazığ, Türkiye

**Keywords:** Breast neoplasms, Diffusion magnetic resonance imaging, Ki-67 antigen, Lymphatic metastasis, Apparent diffusion coefficient, Cortical thickness, Axillary lymph nodes, Breast MRI

## Abstract

**Introduction:**

Accurate preoperative assessment of axillary lymph node metastasis is essential for treatment planning in breast cancer patients. This study aimed to evaluate the diagnostic performance of quantitative MRI parameters, including lymph node morphology and diffusion-weighted imaging-derived Apparent Diffusion Coefficient (ADC) values, in predicting axillary lymph node metastasis.

**Methods:**

This retrospective study included 216 female patients with histopathologically confirmed breast cancer who underwent preoperative breast MRI. Lymph node long and short axis diameters, cortical thickness, ADC values, and derived ratios were measured. Receiver Operating Characteristic (ROC) analysis and multivariate logistic regression were performed to identify independent predictors of lymph node metastasis.

**Results:**

Metastatic lymph nodes demonstrated significantly greater cortical thickness and lower ADC values compared with non-metastatic nodes (*p* < 0.001). Among all parameters, the ADC/cortical thickness ratio showed the highest diagnostic performance (AUC = 0.978; 95% CI: 0.959-0.997), with 96.2% sensitivity and 92.8% specificity. The ADC/cortical thickness ratio successfully detected 128 out of 133 metastatic lymph nodes and 77 out of 83 non-metastatic lymph nodes, with total accuracies of 96.2% and 92.8%, respectively. The test demonstrated a positive predictive value of 95.5% and a negative predictive value of 94.2%. Cortical thickness (>3.5 mm), ADC value (≤900 ×10^−3^ mm^2^/s), and Ki-67 index (≥20%) were identified as independent predictors of lymph node metastasis.

**Discussion:**

The present study demonstrates that lymph node ADC values and ADC-based ratios provide significant diagnostic performance in differentiating metastatic from non-metastatic axillary lymph nodes. The identified cut-off values yielded clinically meaningful sensitivity and specificity, supporting the role of diffusion-weighted imaging as a non-invasive adjunct tool in preoperative axillary evaluation. These findings may contribute to improved patient stratification and may help reduce unnecessary invasive procedures in selected cases.

**Conclusion:**

Quantitative MRI parameters combining morphologic and diffusion features demonstrate high diagnostic accuracy for predicting axillary lymph node metastasis in breast cancer patients. The ADC/cortical thickness ratio is a simple, reproducible imaging biomarker that may support preoperative axillary evaluation and clinical decision-making in routine breast MRI practice.

## 1 INTRODUCTION

Breast cancer stands as the leading cancer type among women, resulting in substantial death rates [1]. The sensitivity of traditional imaging methods like mammography and ultrasound remains low in young women with dense breast tissue, but MRI shows better performance for detecting small and invasive lesions due to its high contrast resolution [2, 3] in the study by Shahbazi-Gahrouei *et al*. The presentation highlighted how advanced MRI techniques, including Diffusion-Weighted Imaging (DWI), Diffusion Tensor Imaging (DTI), and dynamic contrast-enhanced MRI, help diagnose breast cancer and identify its subtypes, and that DWI proved useful for differentiating malignant from benign lesions without contrast agents [3]. In the detection of lymph node metastasis, there is a growing need for more effective imaging approaches that integrate both morphological and advanced MRI-based techniques. While MRI has shown promising results in evaluating breast lesions, its role in specific conditions, such as microcalcification assessment, and in emerging imaging strategies remains under investigation, highlighting the need for further refinement of imaging-based diagnostic tools [4, 5].

In the current literature, the diagnostic accuracy of breast MRI in different tumor subtypes, particularly in young and high-risk groups, and the clinical benefits of new techniques have not been fully established [1-3]. Additionally, it is emphasized that larger-scale studies and real-world data are needed to make MRI-based diffusion methods (DWI and DTI) definitive clinical standards for breast cancer diagnosis and treatment response evaluation [3]. The accuracy and clinical utility of artificial intelligence and deep learning-based approaches have also been investigated for breast cancer diagnosis and lymph node metastasis prediction; however, their clinical applicability remains variable, and further validation is required before routine implementation [5-8].

The main research question of this study investigates whether magnetic resonance imaging quantitative measurements can effectively predict axillary lymph node metastasis in breast cancer patients. It is predicted that ADC values from diffusion-weighted imaging, together with lymph node morphological features and their integration, will achieve high diagnostic precision in differentiating metastatic from non-metastatic lymph nodes. This study also hypothesizes that there is an inverse relationship between ADC values and Ki-67 levels, a tumor proliferation marker, and that this relationship is more pronounced in metastatic lymph nodes. The research objective aims to evaluate MRI-based quantitative parameters (ADC value, cortical thickness, lymph node sizes, and their ratios) for their predictive value of lymph node metastasis in breast cancer patients and to determine appropriate threshold values and independent risk factors for lymph node metastasis.

## 2 METHODS

### 2.1 Study Population and Sample

The research involved patients who received breast magnetic resonance imaging at our hospital's Department of Radiology from January 2017 through December 2024 andreceived a breast cancer diagnosis through histopatho-logical examination. All patients included in the study were female. The sample size was calculated based on an assumed lymph node metastasis prevalence of 60% [9], with a 95% confidence interval and 80% power, requiring a minimum of 200 patients. A total of 216 female patients were included in the study. The median age of the patients was 41 years (range: 24-78 years). The inclusion criteria consisted of a histopathologically confirmed breast cancer diagnosis in female patients, preoperative contrast-enhanced breast MRI images availability, and histopathologically confirmed axillary lymph node status and absence of artifacts in the images. The exclusion criteria consisted of patients who received neoadjuvant chemotherapy, lacked preoperative MRI images, had benign breast lesions, or had insufficient image quality. Male breast cancer patients were not included in this study due to their rarity and potential differences in lymph node metastasis patterns.

Sample size estimation was performed based on an expected prevalence of axillary lymph node metastasis of approximately 60%, a 95% confidence level, and 80% statistical power to detect significant differences in quantitative MRI parameters between metastatic and non-metastatic lymph nodes. The calculated minimum required sample size was 200 patients, and the final cohort of 216 patients was considered sufficient for ROC analysis and multivariate logistic regression.

It should be noted that this study was designed as a diagnostic accuracy study rather than an artificial intelligence classification model. All included patients had histopatho-logically confirmed breast cancer, and the study aimed to evaluate the diagnostic performance of quantitative MRI parameters in discriminating between metastatic and non-metastatic axillary lymph nodes. The research participants were divided into two groups based on axillary lymph node status: 133 patients (61.6%) with metastasis and 83 patients (38.4%) without metastasis.

The sample sizes used for intra-observer (n = 30) and inter-observer (n = 50) reliability analyses were selected in accordance with commonly accepted methodological recommendations for Intraclass Correlation Coefficient (ICC) analysis, which suggest that a minimum of 30 subjects is sufficient for reliable estimation [10].

### 2.2 Operational Definitions

The definition of lymph node metastasis required histopathological evidence of malignant cells in one or more lymph nodes found in the surgical specimen. Cortical thickness was defined as the measurement in millimeters of the thickest cortical region of the lymph node. The ADC value was defined as the apparent diffusion coefficient (×10^−3^ mm^2^/s) obtained from the region of interest in diffusion-weighted imaging.

### 2.3 Imaging Protocol

All MRI examinations were performed using a 1.5 Tesla MRI scanner (Gyroscan Intera, Philips Medical Systems, Best, The Netherlands) with a standard breast coil. Patients were imaged in the prone position using a bilateral breast coil. The standard protocol included axial T1-weighted (TR/TE: 450/10 ms), axial fat-suppressed T2-weighted (TR/TE: 4000/70 ms), diffusion-weighted imaging (b-values: 0, 800 s/mm^2^), and contrast-enhanced dynamic T1-weighted imaging (0.1 mmol/kg gadolinium-DTPA, 2 mL/s injection rate, followed by 20 mL saline flush) sequences.

### 2.4 Image Analysis and Measurements

**Fig. (1):**
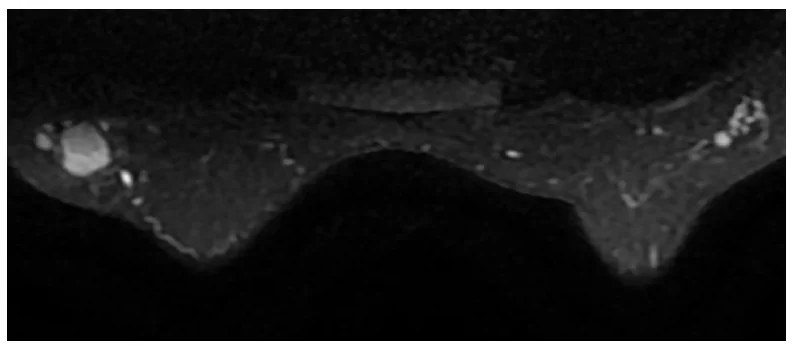
Schematic illustration of standardized lymph node measurement methodology on an axial T2-weighted MR image. The diagram demonstrates the measurement technique for the long-axis diameter (17 mm in this metastatic example), the short-axis diameter (10 mm), and the cortical thickness (7.4 mm), with the calculation of the long-to-short axis ratio (1.7).

**Fig. (2):**
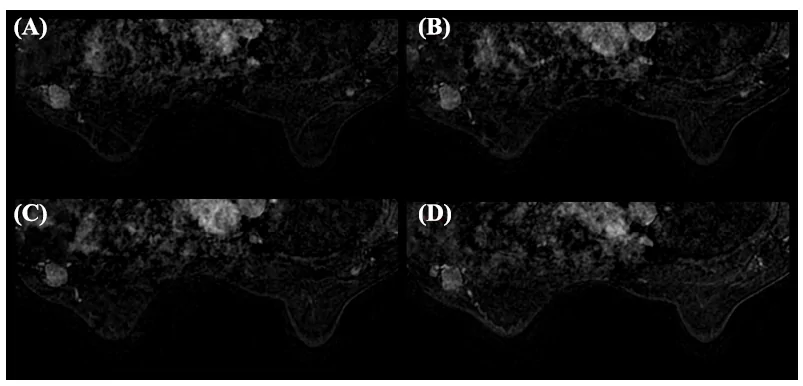
Representative axial T2-weighted fat-suppressed MR images showing morphological characteristics of axillary lymph nodes across four examples. (**A**) Non-metastatic lymph node with preserved fatty hilum and thin cortex (cortical thickness: 2.4 mm). (**B**) Metastatic lymph node with thickened cortex (cortical thickness: 7.4 mm) and loss of fatty hilum. (**C**) Another metastatic lymph node demonstrating cortical thickening (8.7mm). (**D**) Non-metastatic lymph node with normal appearance and thin cortex (2.9 mm).

**Fig. (3):**
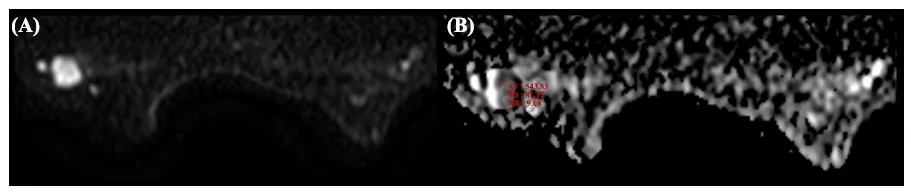
Diffusion-weighted imaging and ADC mapping of axillary lymph nodes. **(A)** DWI (b = 800 s/mm^2^) showing high signal intensity in a metastatic lymph node, indicating diffusion restriction. **(B)** Corresponding ADC map with ROI placement demonstrating low ADC value (747 ×10^−3^ mm^2^/s) in the same metastatic node.

Lymph node sizes, cortical thickness measurements, and ADC values were evaluated by two experienced radiologists (10 and 15 years of breast imaging experience) by consensus. The long and short axis measurements of the lymph nodes were measured in millimeters on axial T2-weighted slices (Fig. **1**). The cortical thickness measurements were performed in millimeters at the thickest cortical region in fat-suppressed T2-weighted images (Fig. **2**). The ADC measurements were obtained by placing a 3-5 mm^2^ circular ROI in the widest section of the lymph node, centered on the central portion, and recording the average ADC values (Fig. **3**). For the pectoral muscle reference measurement, an ROI of 10 mm^2^ was placed in the pectoral muscle in the same section, and the ADC value was measured. The measurements enabled the calculation of all ratio parameters, including the ADC/pectoral muscle ratio, ADC/cortical thickness ratio, and long/short axis ratio. During ADC measurements, regions of interest were carefully placed to avoid necrotic, cystic, or hemorrhagic areas of the lymph nodes, and measurements were obtained from the most homogeneous solid portions.

### 2.5 Observer Agreement

To assess intra-observer reliability, images of 30 randomly selected patients were re-evaluated by the first radiologist 4 weeks later [10]. The intra-observer agreement coefficient (intraclass correlation coefficient, ICC) was found to be 0.92 for cortical thickness and 0.89 for ADC measurements. Inter-observer agreement was assessed by having two radiologists independently evaluate images from 50 patients, and ICC values were 0.88 for cortical thickness and 0.85 for ADC.

### 2.6 Histopathological Evaluation

The histopathological data were obtained from patient records through a retrospective analysis. The recorded parameters included patient age, histological type (invasive ductal or lobular carcinoma), estrogen receptor (ER) status (positive: ≥1%), progesterone receptor (PR) status (positive: ≥1%), HER2 status (IHC 3+ or FISH positive), and Ki-67 proliferation index (%) were included, in line with previously reported clinicopathological classification approaches in breast cancer research [11]. Triple-negative patients were defined as those with all three markers (ER, PR, and HER2) negative.

### 2.7 Statistical Analysis

Statistical analyses were performed using SPSS 25.0 (IBM Corp., Armonk, NY, USA) software. The normality of continuous variables was assessed using the Kolmogorov-Smirnov test. The study used median and interquartile range values to show that the continuous variables did not follow a normal distribution, and the Mann-Whitney U test was used for intergroup comparisons. Categorical variables were expressed as numbers and percentages and analyzed using the chi-square test.ROC (Receiver Operating Characteristic) analysis was performed to evaluate the diagnostic performance of the parameters, and the area under the curve (AUC), sensitivity, specificity values, and optimal threshold values were calculated using the Youden index, a widely accepted method for determining optimal diagnostic cutoff values in ROC analysis [12]. Positive predictive value (PPV) and negative predictive value (NPV) were also calculated to provide category-specific diagnostic performance for each lymph node status group. The research used Spearman correlation analysis to study the relationship between Ki-67 and ADC values. The ADC value distribution between groups was displayed through box plots. The backward stepwise method of multivariate logistic regression analysis identified independent factors predicting lymph node metastasis. The model included the following variables: age, ER negativity, Ki-67 level (≥20% *vs*. <20%), ADC value (≤900 *vs*. >900 ×10^−3^ mm^2^/s), and cortical thickness (≥3.5 *vs*. ≤3.5 mm). Odds ratio values were presented with 95% confidence intervals. The study used *p*-values less than 0.05 to determine statistical significance.

### 2.8 Ethical Considerations

This study was approved by the Clinical Research Ethics Committee of Adiyaman University Faculty of Medicine, Adiyaman, Turkey (Protocol No: 2024/7-11, Date: 17.09.2024). The Good Clinical Practice Guidelines, together with the Declaration of Helsinki, serve as the basis for the ethics committee's operations. The institutional ethics committee waived the need for patient consent because the study used anonymized data from a retrospective design. The researchers maintained patient privacy through data coding for complete anonymity protection. The research team used study-specific identification numbers to replace all patient identifiers after removing personal information. The study team maintained data security through encrypted computer systems and database access restrictions. The study data will remain accessible for at least 5 years after publication, in accordance with institutional data storage regulations. The study was reported in accordance with the STARD (Standards for Reporting of Diagnostic Accuracy Studies) guidelines.

## 3 RESULTS

A total of 216 breast cancer patients were included in the study. Lymph node metastasis was detected in 133 (61.6%) patients, while 83 (38.4%) patients did not have lymph node metastasis. It should be clarified that this is a diagnostic accuracy study rather than an artificial intelligence-based classification model. All 216 patients included in this study had histopathologically confirmed breast cancer, and the study population was divided into two groups based on axillary lymph node status: metastatic (n=133, 61.6%) and non-metastatic (n=83, 38.4%). There was no significant difference in age between the two groups. The median age of lymph node-positive patients was 42 years, while it was 40 years in lymph node-negative patients (Table **1**).

The analysis of hormonal receptor status showed that estrogen receptor positivity was more common in lymph node-negative patients, but this difference did not reach statistical significance. The lymph node-negative group showed higher progesterone receptor positivity, but this difference did not reach statistical significance. The HER2 positivity rates were similar in both groups. However, the Ki-67 proliferation index was significantly higher in the lymph node-positive group. The median Ki-67 value was 20% in patients with lymph node metastasis, while it was 15% in those without metastasis. There were no differences between the groups in terms of histological type, and invasive ductal carcinoma was the predominant histological type in both groups (Table **1**).

When the imaging characteristics of lymph nodes were evaluated, metastatic lymph nodes were found to be signifi-cantly larger in size. The median long-axis value in metastatic lymph nodes was 17 mm, and the median short-axis value was 10 mm, whereas these values were 7 mm and 5 mm, respectively, in non-metastatic lymph nodes. The long/short axis ratio was also higher in the metastatic group. The most prominent difference was observed in cortical thickness measurements. The median cortical thickness in metastatic lymph nodes was 7.4 mm, while it was only 2.4 mm in the non-metastatic group (Table **2**).

Significant differences were observed between the groups in terms of ADC values. ADC values were significantly lower in metastatic lymph nodes. The median ADC value in the metastatic group was 747×10^−3^ mm^2^/s, whereas it was 1002×10^−3^ mm^2^/s in the non-metastatic group. Pectoral muscle ADC values were also lower in the metastatic group, although this difference was not as pronounced as in ADC values. The ADC/pectoral muscle ratio was calculated as 0.49 in the metastatic group and 0.60 in the non-metastatic group. The ADC/cortical thickness ratio showed the highest difference between the groups, with values of 106 in the metastatic group and 417 in the non-metastatic group (Table **2**).

When the relationship between tumor receptor status and lymph node metastasis was examined, a Ki-67 proliferation index of 20% or higher showed a significant association with lymph node metastasis. In 58.6% of lymph node-positive patients, the Ki-67 value was 20% or higher, whereas in lymph node-negative patients, this rate was 42.2%. Although the number of triple-negative patients was higher in the lymph node-positive group, this difference did not reach statistical significance (Table **3**).

**Fig. (4):**
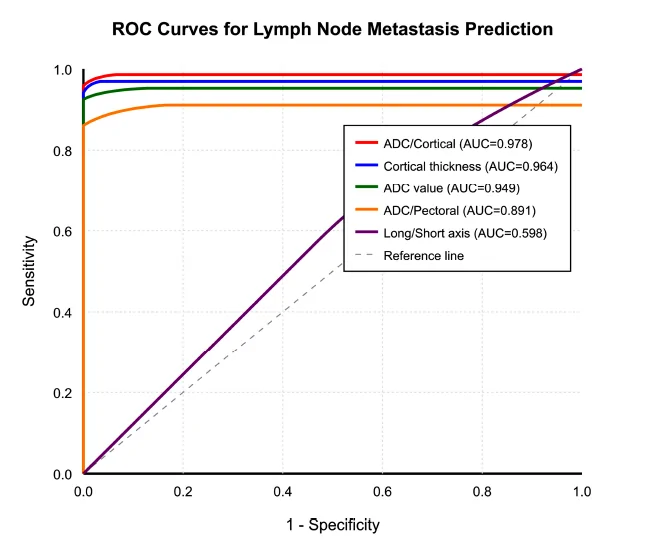
ROC curves for predicting lymph node metastasis: Comparison of MRI-based parameters including ADC/cortical thickness ratio (red), cortical thickness (blue), ADC value (green), ADC/pectoral muscle ratio (orange), and long/short axis ratio (purple). ADC = apparent diffusion coefficient; AUC = area under the curve.

ROC analysis results showed that the ADC/cortical thickness ratio had the highest diagnostic value in predicting lymph node metastasis. The calculated area under the curve for this parameter was 0.978, and a threshold value of 250 yielded a sensitivity of 96.2% and a specificity of 92.8%. The diagnostic performance of cortical thickness measurements proved to be very high. The system reached 95.5% sensitivity and 96.4% specificity when using a threshold value of 3.5 mm. The ADC value was determined using a threshold of 900×10^−3^ mm^2^/s, resulting in 93.2% sensitivity and 85.5% specificity. The ADC/pectoral muscle ratio proved useful for diagnosis, but the long/short-axis ratio showed limited diagnostic value (Table **4** and Fig. **4**).

The researchers determined diagnostic performance for each category by performing individual accuracy assessments for all lymph node status groups. The model detected 128 out of 133 metastatic lymph nodes with 96.2% sensitivity, and 77 out of 83 non-metastatic lymph nodes with 92.8% specificity, using a threshold of ≤250 for the ADC/cortical thickness ratio. The model yielded a positive predictive value (PPV) of 95.5% and a negative predictive value (NPV) of 94.2% according to this study. The >3.5 mm threshold for cortical thickness measurement correctly identified 127 of 133 metastatic nodes and 80 of 83 non-metastatic nodes, achieving a PPV of 97.7% and an NPV of 93.0%. The ADC value correctly identified 124 out of 133 metastatic nodes and 71 out of 83 non-metastatic nodes at the ≤900 ×10^−3^ mm^2^/s threshold. The results indicated 93.2% accuracy for metastatic nodes and 85.5% accuracy for non-metastatic nodes with a positive predictive value of 91.2% and a negative predictive value of 88.8% (Table **4**).

**Fig. (5):**
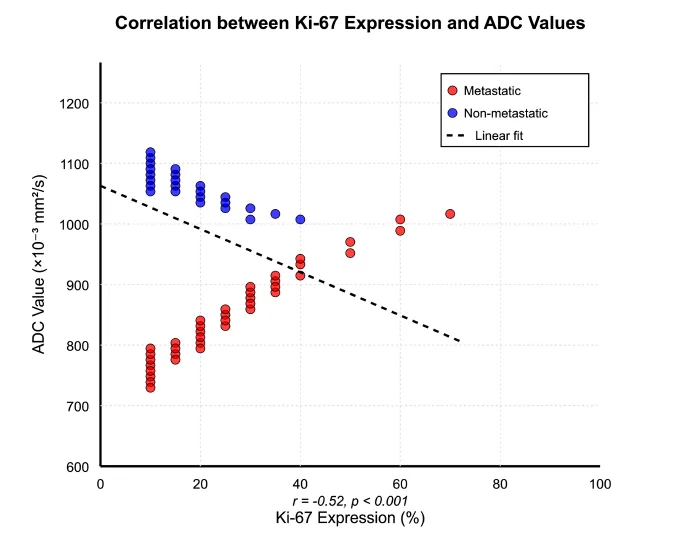
The scatter plot shows an inverse relationship between Ki-67 proliferation index and ADC values through red circles representing metastatic lymph nodes (n=133) and blue circles representing non-metastatic lymph nodes (n=83). The dashed line indicates the linear regression fit.ADC = apparent diffusion coefficient.Spearman correlation coefficient r = -0.52, *p* < 0.001

A negative correlation was found between Ki-67 prolife-ration index and ADC values. As Ki-67 values increased, ADC values decreased. This inverse relationship was observed in both metastatic and non-metastatic lymph nodes, but it was more pronounced in the metastatic group. The correlation coefficient was -0.52, and the relationship was statistically significant (Fig. **5**).

**Fig. (6):**
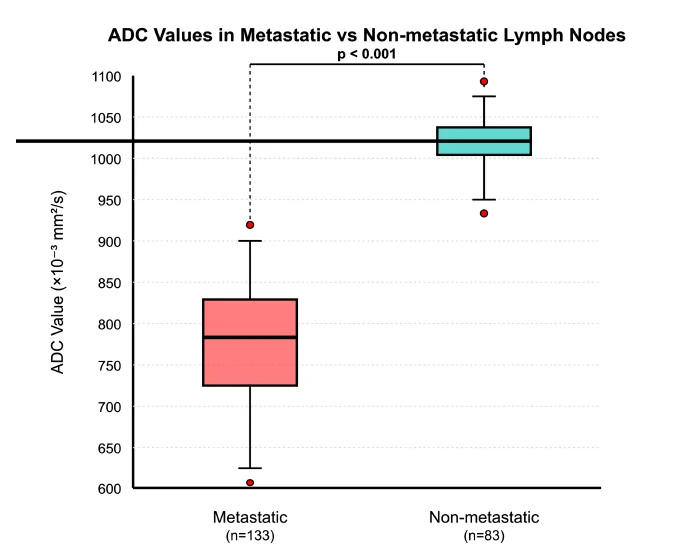
Box plots comparing ADC values between metastatic and non-metastatic lymph nodes: The boxes represent interquartile ranges with median values indicated by horizontal lines. Whiskers extend to the minimum and maximum values, with outliers shown as individual points. ADC = Apparent Diffusion Coefficient. Mann-Whitney U test, *p* < 0.001.

The differences in ADC values between metastatic and non-metastatic lymph nodes were clearly seen in the box plot. The ADC values of metastatic lymph nodes were significantly lower and distributed over a narrower range, whereas those of non-metastatic lymph nodes were higher and more homogeneous (Fig. **6**).

The multivariate logistic regression analysis showed that cortical thickness and ADC value are independent factors that predict lymph node metastasis. A cortical thickness of more than 3.5 mm increased the risk of lymph node metastasis by 127.84-fold, while an ADC value of less than 900×10^−3^ mm^2^/s increased this risk by 18.73-fold. The study found that a Ki-67 level of 20% or higher independently predicted lymph node metastasis, with a 1.95-fold increased risk. The research did not identify Age and ER negativity as independent predictors (Table **5**).

## 4 DISCUSSION

In this study, magnetic resonance imaging was used to evaluate axillary lymph node metastasis in patients with breast cancer. Our findings demonstrate that diagnostic accuracy improves when lymph node morphological features are combined with diffusion-weighted imaging parameters, supporting their potential role as adjunctive tools in clinical assessment.

Lower ADC values observed in metastatic lymph nodes in our cohort are consistent with previous reports in the literature. Prior studies have similarly demonstrated reduced ADC values in metastatic axillary lymph nodes, which is generally attributed to increased cellular density and reduced extracellular space in malignant tissue [13].

Although the diagnostic performance of ADC alone has shown variability across studies, recent evidence supports its role as a useful predictor when applied to solid nodal structures. While some investigations have reported limited performance of ADC in breast lesion characterization [14, 15], other studies have identified ADC as an independent predictor of axillary lymph node metastasis, particularly when incorporated into multivariate models [13, 16]. Our findings further support the contribution of ADC to nodal metastasis prediction in this context.

Cortical thickness emerged as a strong morphological predictor of lymph node metastasis, in agreement with previous ultrasound- and MRI-based studies. Multiple investigations have consistently reported significantly greater cortical thickness in metastatic compared with non-metastatic lymph nodes, underscoring the importance of nodal cortical morphology in axillary evaluation [17-19].

Our research established 3.5 mm as the optimal cortical thickness threshold, achieving 95.5% sensitivity and 96.4% specificity for diagnostic purposes. The system correctly identified 127 metastatic lymph nodes and 80 non-metastatic lymph nodes from the 133 metastatic and 83 non-metastatic nodes. The system achieved a Positive Predictive Value (PPV) of 97.7% and a Negative Predictive Value (NPV) of 93.0% with this threshold. This sensitivity value is significantly higher than the 63.2% sensitivity reported by Chen and colleagues for a 3 mm threshold value [20]. Cho and colleagues reported 60% sensitivity and 89.5% specificity with a 4.35 mm threshold [18]. Loonis and colleagues studied positive predictive values (PPV) at different threshold values and found that PPV increased with increasing cortical thickness; they reported PPV of 63% for 3.5 mm, 67% for 4 mm, and 74% for 4.25 mm [17]. The research study achieved a 97.7% PPV, surpassing the findings reported by Loonis *et al*., and this may be attributed to differences in patient selection criteria and measurement standardization. Our higher sensitivity and specificity values are likely due to the homogeneity of our patient population and standardized measurement techniques [17].

A particularly striking finding was that cortical thickness greater than 3.5 mm increased the risk of metastasis by 127.84-fold in multivariate analysis. Kurt and colleagues also identified cortical thickness as an independent predictor in multivariate analysis, reporting an odds ratio of 16.72 [19]. The high odds ratio observed in our study further supports the role of cortical thickness as a strong adjunctive predictor and may partly explain the excellent diagnostic performance observed in our cohort.

The superior diagnostic performance of the ADC/cortical thickness ratio observed in our cohort supports the growing emphasis on multiparametric imaging approaches for predicting axillary lymph node metastasis. In our study, this ratio demonstrated markedly higher diagnostic accuracy than diffusion parameters alone, highlighting the benefit of integrating morphologic and diffusion-based features. The ADC/cortical thickness ratio at the ≤250 threshold successfully detected 96.2% of the 133 metastatic lymph nodes and 92.8% of the 83 non-metastatic lymph nodes, achieving a positive predictive value of 95.5% and a negative predictive value of 94.2%. The proposed parameter delivers dependable diagnostic results, which show its effectiveness for both lymph node status groups according to these category-specific findings.

Previous studies have reported that ADC values alone may be insufficient for reliable prediction of nodal metastasis, whereas multiparametric approaches improve diagnostic performance [21]. In this context, the higher diagnostic accuracy achieved by the ADC/cortical thickness ratio in our study underscores the added value of combining cortical morphology with diffusion restriction. The researchers established quantitative parameters that achieved high diagnostic accuracy to identify both metastatic and non-metastatic cases effectively for clinical diagnosis.

Although deep learning and radiomic models have shown promising results in axillary lymph node evaluation, reported diagnostic accuracies vary across studies [22-24]. While direct comparisons are not possible due to differences in study design and datasets, the higher diagnostic performance observed in our cohort suggests that simple quantitative ratios derived from routine MRI measurements may achieve diagnostic accuracy within a similar range, while offering advantages in transparency and clinical applicability. The research design focused on diagnostic accuracy assessment of MRI quantitative measurements rather than using artificial intelligence for classification; therefore, researchers should approach machine learning model comparisons with caution.

In addition, normalization strategies such as the ADC/pectoral muscle ratio further support the role of quantitative diffusion-based parameters in improving diagnostic confidence. The ADC/pectoral muscle ratio at the ≤0.56 threshold showed 88.7% sensitivity and 81.9% specificity, enabling researchers to detect 118 out of 133 metastatic and 68 out of 83 non-metastatic lymph nodes (PPV: 88.7%, NPV: 88.3%).

The strong inverse relationship between ADC values and the Ki-67 proliferation index observed in our study is consistent with previous reports and supports the biological link between diffusion restriction and increased tumor cellularity [25, 26]. Although conflicting results have been described in specific lesion subtypes, our findings in axillary lymph nodes reinforce the principle that water diffusivity decreases as proliferative activity increases in solid metastatic tissue.

Furthermore, the association between higher Ki-67 levels and increased risk of lymph node metastasis observed in our cohort aligns with prior studies linking elevated Ki-67 expression to lymphovascular invasion and aggressive tumor behavior [27, 28].

The median Ki-67 value of 20% in our patients with metastatic lymph nodes and 15% in the non-metastatic group suggests that the proliferation index is a valuable marker for predicting lymph node involvement. The ADC value measured by Mounir and colleagues in patients with lymphovascular invasion was 0.735×10^−3^ mm^2^/s [28], which is very close to the 747×10^−3^ mm^2^/s value we found in metastatic lymph nodes. The parallelism indicates that tumors with high proliferative activity are at high risk of both lymphovascular invasion and lymph node metastasis, as reflected in decreased ADC values [28].

From a clinical perspective, the quantitative MRI parameters evaluated in this study—particularly the ADC/cortical thickness ratio—may serve as useful adjunctive tools for preoperative axillary assessment in breast cancer patients. By integrating morphologic and diffusion-based information, these measurements may improve risk stratification and support surgical planning in routine breast MRI practice. Importantly, these findings should be interpreted as complementary imaging information and are not intended to replace established invasive staging procedures, such as sentinel lymph node biopsy. Further prospective multicenter studies are warranted to validate these results and to clarify their role within multidisciplinary decision-making algorithms.

## 5 LIMITATIONS

This study has several limitations that should be acknowledged. First, its retrospective single-center design may limit the generalizability of the findings to different patient populations and clinical settings. Selection bias may also be present, as only patients who underwent preoperative MRI and did not receive neoadjuvant therapy were included.

Second, all measurements were performed by radiologists using a consensus-based approach, which may not fully reflect inter-reader variability encountered in routine clinical practice. Although inter- and intra-observer agreement were assessed, these analyses were conducted in relatively small subsets and may not be representative of all institutions.

Third, imaging was performed using a 1.5 Tesla MRI system, and results may differ with 3.0 Tesla scanners, which offer higher spatial resolution and potentially different ADC values. In addition, manual ROI placement may introduce measurement variability despite careful avoidance of necrotic or non-representative areas.

An important limitation of this study is the lack of external validation. As all analyses were conducted within a single-center cohort, the proposed cutoff values and diagnostic performance may not be directly applicable to other institutions with different imaging protocols or MRI systems. Therefore, these results should be interpreted with caution until validated in independent, multicenter datasets.

Another limitation is the potential risk of model overfitting. Although multicollinearity was assessed and found acceptable, the logistic regression model was developed without internal validation techniques, such as bootstrapping or split-sample validation. Consequently, the stability and reproducibility of the estimated odds ratios and cutoff-dependent predictors require confirmation in external cohorts.

Finally, continuous variables were dichotomized using cutoff values derived from ROC analysis and the Youden index. As these thresholds were not externally validated, they should be considered cohort-specific and should not be generalized without further validation.

Future studies should focus on prospective multicenter validation using standardized imaging protocols, automated or AI-assisted measurement techniques, and correlation with long-term outcomes, such as disease-free and overall survival, to establish the clinical utility of quantitative MRI biomarkers in axillary lymph node evaluation.

## CONCLUSION

This study demonstrated that quantitative MRI parameters can be used to predict axillary lymph node metastasis in breast cancer patients. The combination of cortical thickness and lymph node diffusion properties achieved high diagnostic performance, which would assist clinical decision-making. The ADC/cortical thickness ratio produced exceptional diagnostic results, with an AUC of 0.978, successfully detecting 96.2% of metastatic and 92.8% of non-metastatic lymph nodes (PPV: 95.5%, NPV: 94.2%). This diagnostic accuracy is within the range reported for computational and artificial intelligence-based models in the current literature. The research shows that basic MRI measurements enable doctors to obtain dependable non-invasive markers for lymph node evaluation, which may contribute to improved preoperative risk stratification of axillary lymph node status and potentially reduce unnecessary axillary interventions in selected patients. Importantly, these findings should be interpreted as supportive imaging information and are not intended to replace established invasive staging procedures, such as sentinel lymph node biopsy. The negative relationship between ADC values and Ki-67 proliferation index confirms the biological origin of our imaging results because diffusion restriction directly correlates with cellular growth in metastatic nodes. The implementation of these parameters within standard breast MRI protocols would lead to better preoperative staging results and improved treatment planning for breast cancer patients.

## Figures and Tables

**Table 1:** Demographic and clinical characteristics.

**Characteristics**	**Lymph Node Positive (n=133)**	**Lymph Node Negative (n=83)**	***p*-value**
**Age, years**	42.0 [38.0-54.0]	40.0 [36.0-55.0]	0.472
**ER positive, n (%)**	102 (76.7)	70 (84.3)	0.173
**PR positive, n (%)**	58 (43.6)	47 (56.6)	0.062
**HER2 positive, n (%)**	15 (11.3)	10 (12.0)	0.859
**Ki-67, %**	20.0 [10.0-35.0]	15.0 [10.0-25.0]	0.038
**Histological type**	-	-	0.654
**Invasive ductal**	125 (94.0)	77 (92.8)	-
**Lobular**	8 (6.0)	6 (7.2)	-

**Note:** Data are presented as median [interquartile range] or n (%). ᵃMann-Whitney U test, ᵇChi-square test; ER: Estrogen receptor, PR: Progesterone receptor, HER2: Human epidermal growth factor receptor 2, Ki-67: Cellular proliferation marker; *p* < 0.05 is considered statistically significant.

**Table 2:** Lymph node imaging characteristics.

**Parameters**	**Lymph Node Positive (n=133)**	**Lymph Node Negative (n=83)**	***p*-value**
**Long axis, mm**	17.0 [13.0-22.0]	7.0 [6.0-10.0]	<0.001
**Short axis, mm**	10.0 [7.0-13.0]	5.0 [4.0-6.0]	<0.001
**Long/short axis ratio**	1.86 [1.50-2.14]	1.60 [1.33-1.83]	0.015ᵃ
**Cortical thickness, mm**	7.4 [5.2-8.7]	2.4 [2.3-2.9]	<0.001
**ADC value (×10^−3^ mm^2^/s)**	747 [682-837]	1002 [987-1031]	<0.001ᵃ
**Pectoral muscle ADC value (×10^−3^ mm^2^/s)**	1576 [1502-1663]	1652 [1583-1712]	0.021
**ADC/Pectoral muscle ratio**	0.49 [0.43-0.55]	0.60 [0.58-0.63]	<0.001
**ADC/Cortical thickness**	106 [85-134]	417 [354-443]	<0.001

**Note:** Data are presented as median [interquartile range]; ᵃMann-Whitney U test; ADC: Apparent diffusion coefficient; ADC values are given as ×10^−3^ mm^2^/s; *p* < 0.05 is considered statistically significant.

**Table 3:** Relationship between tumor receptors and lymph node metastasis.

**Receptor Status**	**Lymph Node Positive n (%)**	**Lymph Node Negative n (%)**	***p*-value**
**ER**	-	-	0.173
**Positive**	102 (76.7)	70 (84.3)	-
**Negative**	31 (23.3)	13 (15.7)	-
**PR**	-	-	0.062
**Positive**	58 (43.6)	47 (56.6)	-
**Negative**	75 (56.4)	36 (43.4)	-
**HER2**	-	-	0.859
**Positive**	15 (11.3)	10 (12.0)	-
**Negative**	118 (88.7)	73 (88.0)	-
**Ki-67**	-	-	0.024
**≥20%**	78 (58.6)	35 (42.2)	-
**<20%**	55 (41.4)	48 (57.8)	-
**Triple negative**	16 (12.0)	6 (7.2)	0.263

**Note:** Data are presented as n (%). ᵇChi-square test; ER: Estrogen receptor, PR: Progesterone receptor, HER2: Human epidermal growth factor receptor 2, Ki-67: Cellular proliferation marker, Triple negative: ER, PR, and HER2 negativity; *p* < 0.05 was considered statistically significant.

**Table 4:** ROC analysis results and category-specific diagnostic performance.

**Parameters**	**AUC**	**95% CI**	**Threshold Value**	**Sensitivity (%)**	**Specificity (%)**	**TP/Total Metastatic**	**TN/Total Non-Metastatic**	**PPV (%)**	**NPV (%)**	***p*-value**
**ADC/Cortical thickness**	0.978	0.959-0.997	≤250	96.2	92.8	128/133	77/83	95.5	94.2	<0.001
**Cortical thickness**	0.964	0.937-0.991	>3.5 mm	95.5	96.4	127/133	80/83	97.7	93.0	<0.001
**ADC value**	0.949	0.919-0.979	≤900	93.2	85.5	124/133	71/83	91.2	88.8	<0.001
**ADC/Pectoral muscle ratio**	0.891	0.847-0.935	≤0.56	88.7	81.9	118/133	68/83	88.7	88.3	<0.001
**Long/short axis ratio**	0.598	0.523-0.673	>1.75	64.7	59.0	86/133	49/83	71.7	51.0	0.015

Abbreviations: ROC: Receiver operating characteristic, AUC: Area under the curve, CI: Confidence interval, ADC: Apparent diffusion coefficient, TP: True positive, TN: True negative, PPV: Positive predictive value, NPV: Negative predictive value; *p* < 0.05 is considered statistically significant.

**Table 5:** Multivariate logistic regression analysis.

**Variables**	**Odds Ratio**	**95% CI**	***p*-value**
**Age**	1.02	0.98-1.06	0.342
**ER negativity**	1.64	0.82-3.28	0.162
**Ki-67 level (≥20% *vs*. <20%)**	1.95	1.08-3.52	0.027
**ADC value (≤900 *vs*. >900)**	18.7	8.64-40.59	<0.001
**Cortical thickness (>3.5 *vs*. ≤3.5 mm)**	127.84	34.85-469.01	<0.001

**Abbreviations:** CI: Confidence Interval, ER: Estrogen Receptor, ADC: Apparent Diffusion Coefficient, Ki-67: Cellular proliferation marker; Multivariate logistic regression analysis; *p* < 0.05 was considered statistically significant.

## Data Availability

The data of current study are available from corresponding author, [E.K], on a reasonable request.

## LIST OF ABBREVIATIONS

ADC: Apparent Diffusion Coefficient
AUC: Area Under the Curve
ROC: Receiver Operating Characteristic
MRI: Magnetic Resonance Imaging
DWI: Diffusion-Weighted Imaging
LN: Lymph Node
ER: Estrogen Receptor
PR: Progesterone Receptor
HER2: Human Epidermal Growth Factor Receptor 2
CI: Confidence Interval
SD: Standard Deviation
OR: Odds Ratio

## STANDARDS OF REPORTING

STARD guidelines were followed.

## References

[1] Yara D., Oroszi T. (2025). Understanding breast cancer: A comprehensive review of epidemiology, risk factors, and treatment strategies.. Adv. Breast Cancer Res..

[2] Alaref A., Hassan A., Sharma Kandel R., Mishra R., Gautam J., Jahan N. (2021). Magnetic resonance imaging features in different types of invasive breast cancer: A systematic review of the literature.. Cureus.

[3] Shahbazi-Gahrouei D., Aminolroayaei F., Nematollahi H., Ghaderian M., Gahrouei S.S. (2022). Advanced magnetic resonance imaging modalities for breast cancer diagnosis: An overview of recent findings and perspectives.. Diagnostics.

[4] Bennani-Baiti B., Baltzer P.A. (2017). MR imaging for diagnosis of malignancy in mammographic microcalcifications: A systematic review and meta-analysis.. Radiology.

[5] Vrdoljak J., Krešo A., Kumrić M., Martinović D., Cvitković I., Grahovac M., Vickov J., Bukić J., Božic J. (2023). The role of AI in breast cancer lymph node classification: A comprehensive review.. Cancers.

[6] Liu C.J., Zhang L., Sun Y., Geng L., Wang R., Shi K.M., Wan J.X. (2023). Application of CT and MRI images based on an artificial intelligence algorithm for predicting lymph node metastasis in breast cancer patients: A meta-analysis.. BMC Cancer.

[7] Ramli Hamid M.T., Ab Mumin N., Wong Y.V., Chan W.Y., Rozalli F.I., Rahmat K. (2023). The effectiveness of an ultrafast breast MRI protocol in the differentiation of benign and malignant breast lesions.. Clin. Radiol..

[8] Abhisheka B., Biswas S.K., Purkayastha B. (2023). A comprehensive review on breast cancer detection, classification and segmentation using deep learning.. Arch. Comput. Methods Eng..

[9] Sopik V., Narod S.A. (2018). The relationship between tumour size, nodal status and distant metastases: On the origins of breast cancer.. Breast Cancer Res. Treat..

[10] Koo T.K., Li M.Y. (2016). A guideline of selecting and reporting intraclass correlation coefficients for reliability research.. J. Chiropr. Med..

[11] Turhan A., Özyurt N. (2025). Molecular subtypes and clinicopathological characteristics of breast cancer across age groups: A retrospective analysis.. J Health Sci Med.

[12] Fluss R., Faraggi D., Reiser B. (2005). Estimation of the Youden index and its associated cutoff point.. Biom. J..

[13] Chen H., Wang X., Huang Y., Cao Y., Cao M., Hu X., Mou F., Gong X., Tang S., Wang L., Li L., Yu T., Cheng Y., Zhang J. (2025). Nomograms integrating MRI-derived apparent diffusion coefficient and clinicopathologic features for prediction of axillary lymph node metastasis in breast cancer.. Radiol. Imaging Cancer.

[14] Lin C.X., Tian Y., Li J.M., Liao S.T., Liu Y.T., Zhan R.G., Du Z.L., Yu X.R. (2023). Diagnostic value of multiple b-value diffusion-weighted imaging in discriminating the malignant from benign breast lesions.. BMC Med Imaging.

[15] Avendano D., Marino M.A., Leithner D., Thakur S., Bernard-Davila B., Martinez D.F., Helbich T.H., Morris E.A., Jochelson M.S., Baltzer P.A.T., Clauser P., Kapetas P., Pinker K. (2019). Limited role of dwi with apparent diffusion coefficient mapping in breast lesions presenting as non-mass enhancement on dynamic contrast-enhanced MRI.. Breast Cancer Res.

[16] Chen Y., Wang J., Zhang X., Yang W., Chen H., Bao B., Qiu Y., Tian L. (2021). Correlation between apparent diffusion coefficient and pathological characteristics of patients with invasive breast cancer.. Ann. Transl. Med..

[17] Loonis A.S.T., Chesebro A.L., Bay C.P., Portnow L.H., Weiss A., Chikarmane S.A., Giess C.S. (2024). Positive predictive value of axillary lymph node cortical thickness and nodal, clinical, and tumor characteristics in newly diagnosed breast cancer patients.. Breast Cancer Res. Treat..

[18] Cho P., Park C.S., Park G.E., Kim S.H., Kim H.S., Oh S.J. (2023). Diagnostic usefulness of diffusion-weighted mri for axillary lymph node evaluation in patients with breast cancer.. Diagnostics.

[19] Kurt N., Binboga Kurt B., Gulsaran U., Uslu B., Celik A.O., Sut N., Tastekin E., Karabulut D., Tuncbilek N. (2022). Diffusion tensor imaging and diffusion-weighted imaging on axillary lymph node status in breast cancer patients.. Diagn. Interv. Radiol..

[20] Chen S.T., Lai H.W., Chang J.H.M., Liao C.Y., Wen T.C., Wu W.P., Wu H.K., Lin Y.J., Chang Y.J., Chen S.T., Chen D.R., Huang H.I., Hung C.L. (2023). Diagnostic accuracy of pre-operative breast magnetic resonance imaging (MRI) in predicting axillary lymph node metastasis: Variations in intrinsic subtypes, and strategy to improve negative predictive value—an analysis of 2473 invasive breast cancer patients.. Breast Cancer.

[21] Song S.E., Woo O.H., Cho Y., Cho K.R., Park K.H., Kim J.W. (2023). Prediction of axillary lymph node metastasis in early-stage triple-negative breast cancer using multiparametric and radiomic features of breast MRI.. Acad. Radiol..

[22] Kong Q., Chen Y., Sui Y., Chen S., Lv X., Tang W., Zhong Z., Yu X., Jiang K., Zhang L., Chen J., Qin J., Guo Y. (2025). The role of multiparametric mri radiomics for preoperative prediction of axillary lymph node metastasis in patients with invasive breast cancer: A comparative study.. Cancer Innov.

[23] Qiu Y., Zhang X., Wu Z., Wu S., Yang Z., Wang D., Le H., Mao J., Dai G., Tian X., Zhou R., Huang J., Hu L., Shen J. (2022). MRI-based radiomics nomogram: Prediction of axillary non-sentinel lymph node metastasis in patients with sentinel lymph node-positive breast cancer.. Front Oncol.

[24] He X., Liao Y., Zhu W., Zhang S., Shi Y., Ren D., Liu L., Zhao X., Ye M., Yang X., Wang G. (2025). Predicting axillary lymph node metastasis in small breast cancers using convolutional neural networks for multiparametric magnetic resonance imaging (MRI).. Clin Radiol.

[25] Tanişman Ö., Kiziltepe F.T., Yildirim Ç., Coşar Z.S. (2023). Prediction of prognostic factors in breast cancer: A noninvasive method utilizing histogram parameters derived from Adc maps.. Heliyon.

[26] Nie T., Feng M., Yang K., Guo X., Yuan Z, Zhang Z, Yan G (2023). Correlation between dynamic contrast-enhanced MRI characteristics and apparent diffusion coefficient with Ki-67-positive expression in non-mass enhancement of breast cancer.. Sci Rep.

[27] Chen H., Meng X., Hao X., Li Q., Tian L., Qiu Y., Chen Y. (2022). Correlation analysis of pathological features and axillary lymph node metastasis in patients with invasive breast cancer.. J Immunol Res.

[28] Mounir A.M., Shokeir F.A., Abd Elraouf G.H. (2025). Breast imaging: Correlation between axillary lymph nodes apparent diffusion coefficient and pathological lymphovascular invasion in patients with invasive breast cancer.. Eur. J. Breast Health.

